# Auto-antibodies targeting brain antigens in *Plasmodium falciparum* infected patients as biomarkers of Cerebral Malaria

**DOI:** 10.1186/1475-2875-9-S2-P4

**Published:** 2010-10-20

**Authors:** Devendra Bansal, Fabien Herbert, Prakash Deshpande, Christophe Bécavin, Vincent Guiyedi, Ilaria de Maria, Pierre-André Cazenave, Gyan Chandra Mishra, Cristiano Ferlini, Constantin Fesel, Arndt Benecke, Sylviane Pied

**Affiliations:** 1 CIIL, INSERM U1019- CNRS UMR 8204, Institut Pasteur de Lille, France; 2 NCCS, Pune, India; 3 IHES, Bures sur Yvettes, France; 4 UCSC, Rome, Italy; 5 IGC, Oeiras, Portugal

## Background

The main processes in the pathogenesis of cerebral malaria caused by *Plasmodium falciparum* involved sequestration of parasitized red blood cells and immunopathological responses. Among immune factors, IgG autoantibodies to brain antigens are increased in *P. falciparum* infected patients and correlate with disease severity in African children. Nevertheless, their role in the pathophysiology of cerebral malaria (CM) is not fully defined. We extended our analysis to an Indian population with genetic backgrounds and endemic and environmental status different from Africa to determine if these autoantibodies could be either a biomarker or a risk factor of developing CM.

## Methods/principal findings

We investigated the significance of these self-reactive antibodies in clinically well-defined groups of *P. falciparum* infected patients manifesting mild malaria (MM), severe non-cerebral malaria (SM), or CM and in control subjects from Gondia, a malaria epidemic site in central India using quantitative immunoprinting and multivariate statistical analyses. We identified beta tubulin III (TBB3) as a novel discriminant brain antigen in the prevalence of CM. In addition, circulating IgG from CM patients highly react with recombinant TBB3. Overall, correspondence analyses based on singular value decomposition show a strong correlation between IgG anti-TBB3 and elevated concentration of cluster-II cytokine (IFNγ, IL1β, TNFα, TGFβ) previously demonstrated to be a predictor of CM in the same population.

## Conclusions/significance

Collectively, these findings validate the relationship between antibody response to brain induced by *P. falciparum* infection and plasma cytokine patterns with clinical outcome of malaria. They also provide significant insight into the immune mechanisms associated to CM by the identification of TBB3 as a new disease-specific marker and potential therapeutic target.

